# Editorial: Nutrition and exercise immunology

**DOI:** 10.3389/fnut.2023.1147518

**Published:** 2023-02-01

**Authors:** David C. Nieman, Glen Davison, Vassilis Mougios, Laurel M. Wentz

**Affiliations:** ^1^Department of Biology, North Carolina Research Campus, Human Performance Laboratory, Appalachian State University, Boone, NC, United States; ^2^School of Sport and Exercise Sciences, Division of Natural Sciences, University of Kent, Canterbury, United Kingdom; ^3^Laboratory of Evaluation of Human Biological Performance, School of Physical Education and Sports Science at Thessaloniki, Aristotle University of Thessaloniki, Thessaloniki, Greece; ^4^Department of Nutrition and Healthcare Management, Appalachian State University, Boone, NC, United States

**Keywords:** sports nutrition, immunology, fruit, dairy, probiotics, almonds

This Research Topic focused on new findings regarding the influence of nutrition-based interventions on exercise-induced perturbations in immunity. The immune system reflects the physiological stress that exercisers experience during demanding exercise workloads. Nutrition-based strategies to mitigate exercise-induced immune-related stresses began with the unexpected discovery during the 1990s that carbohydrate ingestion before and during prolonged and intensive exercise was linked to a sizeable decrease in post-exercise inflammation (1). Numerous other nutritional interventions have been studied within an exercise-immune context, with varied success and efficacy (2–4). A recent focus has been the use of multiomics outcomes to better capture these complex interactions using a human systems biology approach (5, 6). The four papers in this Research Topic include findings from food-based clinical trials with bananas, blueberries, dairy-based beverages, and almonds, and one review paper on probiotics.

A previous study showed that carbohydrate intake (from bananas or a 6% sugar beverage) had a strong effect in reducing plasma oxylipin concentrations after an intensive 75-km cycling bout (7). Oxylipins are bioactive lipid oxidation products that are upstream regulators of inflammation, immunity, and many other physiological processes (8). More than 60 oxylipins (half derived from arachidonic acid, ARA) can be generated during stressful exercise through the metabolism of cell membrane *n-6* and *n-3* polyunsaturated fatty acids (PUFAs) by cyclooxygenase (COX), lipoxygenase (LOX), and cytochrome P450 (CYP) enzyme systems (7, 8). The article, “*Blueberry and/or banana consumption mitigate arachidonic, cytochrome P450 oxylipin generation during recovery from 75-km cycling: a randomized trial*,” extended these findings (Nieman, Gillitt, et al.). This study confirmed the oxylipin-lowering effect of acute carbohydrate ingestion during prolonged and intensive exercise. A novel finding was that 2-week blueberry ingestion (1 cup equivalent/day) increased plasma concentrations of 24 gut-derived phenolic metabolites and decreased post-exercise concentrations of pro-inflammatory ARA-CYP-generated oxylipins. In other words, cyclists exercising vigorously for about 3 h with blueberry gut-derived metabolites in their systems had lower plasma ARA-CYP oxylipins during several hours of recovery. Banana and/or blueberry ingestion did not completely counter post-exercise increases in inflammatory-related oxylipins, but rather attenuated the magnitude of these increases. This study used a multiomics approach to afford a better understanding of the multifaceted interactions between metabolites generated in the gut from blueberry polyphenols and those produced from cell membrane fatty acids.

A study published in 2006 touted chocolate milk as an effective recovery ergogenic aid (9). This discovery was followed by many other similar studies investigating the physiological responses to a variety of dairy milk-based supplements (10). A recent focus has been the potential effect of dairy milk beverages on exercise-induced immune perturbations. The article, “*Does the nutritional composition of dairy milk based recovery beverages influence post-exercise gastrointestinal and immune status, and subsequent markers of recovery optimisation in response to high intensity interval exercise?*”, focused on the effects of flavored dairy-milk recovery beverages of different energy and nutrient densities on gastrointestinal, immune, and metabolic recovery outcomes (Russo et al.). In this crossover study, participants completed 2 h of high-intensity interval running followed by consumption of two different chocolate flavored dairy-milk beverages matched for volume but differing in carbohydrate and protein content. No placebo beverage was included in this study. Exercise-induced changes in several types of outcomes including those related to performance, gastrointestinal integrity, and immune function (e.g., neutrophil degranulation) did not differ between the trials. The authors concluded that ingesting a dairy milk-based supplement beverage with substantially higher vs. lower levels of protein and carbohydrate did not enhance exercise recovery.

Athletic endeavor can be associated with immune dysfunction, an increased risk for acute respiratory infections, and negative gastrointestinal symptoms. Probiotics are a common dietary supplement used to potentially counter these health problems (11). Probiotics are foods or supplements with live microorganisms that are intended to have health benefits when consumed. They can be found in yogurt, other fermented foods, and dietary supplements. The systematic review paper “*Health benefits of probiotics in sport and exercise - non-existent or a matter of heterogeneity? A systematic review*,” summarized data from 41 studies (Heimer et al.). The studies varied so widely in dosing regimens and outcome assessments that the authors were unable to provide conclusive consensus statements. The authors provided a variety of recommendations for future studies including the use of validated questionnaires, improved immune outcomes, and better guidelines for probiotics dosing strategies.

Almonds are a nutrient-dense food with considerable amounts of protein, healthy types of fats, vitamin E, several important minerals, and polyphenols (164 mg/57-g serving). In randomized clinical trials, almond consumption has been related to multiple health, metabolic, and disease prevention benefits (12). The randomized trial presented in the paper, “*Almond intake alters the acute plasma dihydroxy-octadecenoic acid (DiHOME) response to eccentric exercise*,” examined whether or not 4-weeks almonds intake (57 g/day) compared to cereal bars intake would improve recovery from a 90-min “weekend warrior” eccentric exercise bout (Nieman, Omar, et al.). Using a multiomics approach, almond intake was linked to a significant elevation in gut-derived metabolites from almond polyphenols, a post-exercise increase in plasma 12,13-DiHOME, and a decrease in plasma 9,10-DiHOME. 12,13-DiHOME is a lipokine from brown adipose tissue that is elevated in fit and lean individuals and exerts positive effects on metabolic health and energy regulation (13). Other almond-related benefits for exercisers revealed in this study included reduced feelings of fatigue and tension, better leg-back strength during recovery, and decreased muscle damage during the first day of recovery. In general, the data support that depending on the context, nutritional support of sports performance can extend beyond carbohydrate and water and include foods, such as almonds, that supply a wide array of beneficial nutrients.

## Author contributions

DN, GD, VM, and LW wrote this editorial and agree to be accountable for the content of the work. All authors contributed to the article and approved the submitted version.
